# Nicotine Exposure Exacerbates Development of Cataracts in a Type 1 Diabetic Rat Model

**DOI:** 10.1155/2012/349320

**Published:** 2012-09-20

**Authors:** Nima Tirgan, Gabriela A. Kulp, Praveena Gupta, Adam Boretsky, Tomasz A. Wiraszka, Bernard Godley, Ronald G. Tilton, Massoud Motamedi

**Affiliations:** ^1^Department of Ophthalmology and Visual Sciences, University of Texas Medical Branch at Galveston, Galveston, TX 77555, USA; ^2^Center for Biomedical Engineering, University of Texas Medical Branch at Galveston, Galveston, TX 77555, USA; ^3^Endocrinology, Department of Internal Medicine, Division of Endocrinology & Metabolism, University of Texas Medical Branch at Galveston, Galveston, TX 77555, USA

## Abstract

Diabetes and smoking are known risk factors for cataract development. In this study, we evaluated the effect of nicotine on the progression of cataracts in a type 1 diabetic rat model. Diabetes was induced in Sprague-Dawley rats by a single injection of 65 mg/kg streptozotocin. Daily nicotine injections were administered subcutaneously. Forty-five rats were divided into groups of diabetics with and without nicotine treatment and controls with and without nicotine treatment. Progression of lens opacity was monitored using a slit lamp biomicroscope and scores were assigned. To assess whether systemic inflammation played a role in mediating cataractogenesis, we studied serum levels of eotaxin, IL-6, and IL-4. The levels of the measured cytokines increased significantly in nicotine-treated and untreated diabetic animals versus controls and demonstrated a positive trend in the nicotine-treated diabetic rats. Our data suggest the presence of a synergistic relationship between nicotine and diabetes that accelerated cataract formation via inflammatory mediators.

## 1. Introduction

Cataract development affects over 50 million people worldwide leading to a decrease in visual function and a reduction in overall quality of life. Risk factors for cataract development include diabetes, glaucoma, smoking, ocular inflammation, trauma, advanced age, excessive sunlight exposure, and genetics. Numerous studies have demonstrated the prominent role of diabetes as a risk factor in the development of cataracts [1–6]. Clinical studies have also established a positive correlation between smoking and an increased risk of cataract formation [7–11]. Likewise, smokeless tobacco use has a significant association with cataract development [12]. In addition, cataract formation can be a secondary complication of uncontrolled hyperglycemia in diabetics. This is due to posttranslational modifications of lens proteins via nonenzymatic glycation reactions causing clouding of the lens. Various studies have investigated the formation of lens opacities in Streptozotocin- (STZ-) induced diabetic rat models. Zhang et al. 2008 [13] observed initial stages of cataract formation macroscopically 3 weeks after induction of diabetes and whole lens opacity after 12 weeks. Additionally, scanning electron microscopy revealed structural anomalies in diabetic rat lenses as early as one week after STZ injection [14]. Thus, a hyperglycemic environment results in greater susceptibility of ocular tissue to further damage from environmental factors and stress.

Nicotine is a low molecular weight alkaloid that is absorbed by skin contact and inhalation and constitutes a major active component in tobacco products. It is highly lipid soluble and rapidly crosses the blood-brain barrier [15]. Nicotine has been shown to modulate the immune response and mediate inflammatory cell signaling pathways [16]. Additionally, nicotine has been shown to decrease insulin sensitivity following prolonged use of tobacco products or nicotine gum [17]. This may indicate broader implications regarding the use of products containing nicotine for both type 1 and type 2 diabetic patients.

The effect of nicotine on the progression of cataracts in diabetic eyes has not been investigated to date. The purpose of this study was to examine cataract formation in an established type 1 diabetic rat model under the influence of chronic nicotine exposure. The results of this study may shed light on the increased risk of cataract formation associated with the use of tobacco products in the diabetic population.

## 2. Materials and Methods

### 2.1. Animal Protocol

We adhered to the ARVO (Association for Research in Vision and Ophthalmology) statement and NIH guidelines for Use of animals in ophthalmic and vision research. Animal use protocol was approved by the UTMB Institutional Animal Care and use Committee. Male Sprague-Dawley rats were purchased from Jackson Laboratories (Bar Harbor, Maine) at a weight of 200 to 250 g and acclimated for one week with a 12-hour light/dark cycle, constant temperature and humidity, and ad libitum access to food and water. Diabetic rats were housed in individual cages, while nondiabetic animals were housed two per cage.

Body weights and nonfasting blood glucose were recorded weekly throughout the study. A total of 45 rats (*n* = 21 in experiment 1 and *n* = 24 in experiment 2) were used. Animals were placed into 4 groups, including (i) diabetic with nicotine treatment (*n* = 11); (ii) diabetic without nicotine treatment (*n* = 10); (iii) control with nicotine treatment (*n* = 12); (iv) control (*n* = 12). Diabetes was induced with intraperitoneal injection of STZ at 65 mg/kg in ice cold sodium citrate buffer (pH 4.5) [18]. Blood glucose was measured via tail nick using a glucometer 5 days following injection of STZ, and rats that were not hyperglycemic (fed glucose > 300 mg/dL) were excluded from the study. Blood samples were collected at the end of experiments for serum cytokine measurements. 

### 2.2. Nicotine Preparation and Administration

(−)-Nicotine hydrogen tartrate salt (Sigma) was dissolved in phosphate-buffered saline and administered subcutaneously at alternating sites to prevent accumulation of the drug with repeated injection. Three weeks after induction of diabetes, nicotine treatment was initiated at 0.3 mg/kg for 1 week. The dose was increased to 0.9 mg/kg for 3 days and then increased by 0.3 mg/kg intervals every 3 days until a final dose of 2.1 mg/kg was reached and continued for the duration of the experiment. This treatment regimen was developed experimentally to avoid acute toxicity in nicotine-naive animals. 

### 2.3. Slit Lamp Examination and Cataract Scoring

Rat eyes were examined and cataracts were graded at week 9 after initiation of nicotine treatment. In a subset of animals (experiment 2) at weeks 10 and 11, cataract scores were re-evaluated. A slit lamp biomicroscope (Zeiss 30-SLM) was used and the severity of cataract was graded by a masked observer, according to the Oxford classification system [19]: grade 0, clear; grade 1, clear nuclear with wide sutures; grade 2, slight dense nuclear with opacities radiating from sutures; grade 3, dense nuclear without clefts; grade 4, dense nuclear with clefts; grade 5, nuclear cataract with clefts; grade 6, nuclear cataract with dense radial opacities; grade 7, nuclear cataract with whole lens opacities.

Rats were anesthetized with isoflurane and the eyes immobilized by subluxation. Rat pupils were dilated with one drop of 1% tropicamide prior to cataract imaging. Cataract images were captured using a Nikon D70S digital camera. At the end of the study, the animals were euthanized by exsanguination under deep anesthesia with ketamine (100 mg/kg) and xylazine (10 mg/kg) and serum was stored at −80°C until analyzed.

### 2.4. Cytokine Analysis

A panel of cytokines, including Eotaxin, IL-6, and IL-4, that are prominent cytokines involved in inflammation and diabetes, was measured in serum using multiplex assay according to manufacturer's protocols (Millipore, Billerica, MA, USA).

### 2.5. Statistics

One- and 2-way ANOVA with pair wise comparison and Student's *t*-test were used as appropriate. Data are presented as mean ± SEM with significance at *P* < 0.05.

## 3. Results

### 3.1. Body Weights and Blood Glucose

**Figure 1:**
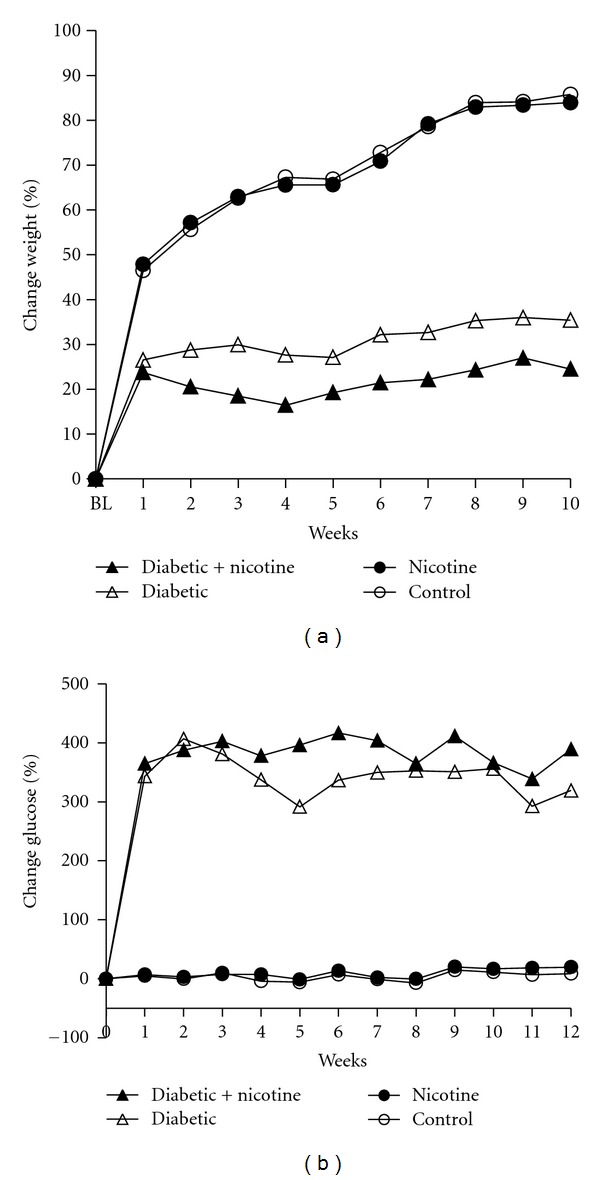
Percent change in body weight (a) and glucose (b) for experimental groups: untreated diabetics (∆), nicotine-treated control (•), nicotine-treated diabetics (▲), and control (°). (a): Both diabetic groups had impaired weight gain, while both nicotine-treated and untreated control groups continued to gain weight throughout the study. The symbol “BL” represents baseline on the *x*-axis. (b): Both treated and untreated diabetic groups had increased blood glucose (mg/dL) as compared to control rates.

Average weekly weight (% change from baseline) per group is presented in Figure 1(a). Both nicotine-treated and untreated diabetic groups continued to gain weight, but at a lower rate than the non-diabetic groups. Although there was a trend for the nicotine-treated diabetics to have lower weights as compared to the untreated diabetics, this finding was not statistically significant (*P* > 0.05). Both nicotine-treated and untreated diabetic groups had increased blood glucose (mg/dL) as compared to control rats (Figure 1(b)).

### 3.2. Cataracts

**Figure 2:**
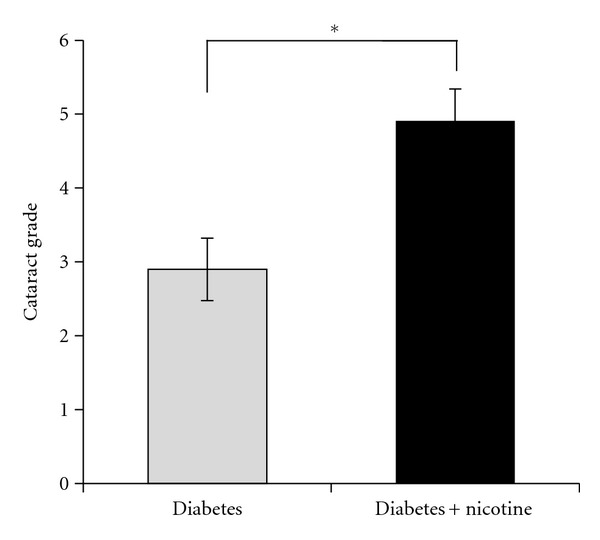
Cataract scores in nicotine-treated and untreated diabetic rats are shown after 9 weeks of treatment. The nicotine-treated diabetic rats had increased cataract scores as compared to untreated diabetics (*P < 0.05).

**Figure 3:**
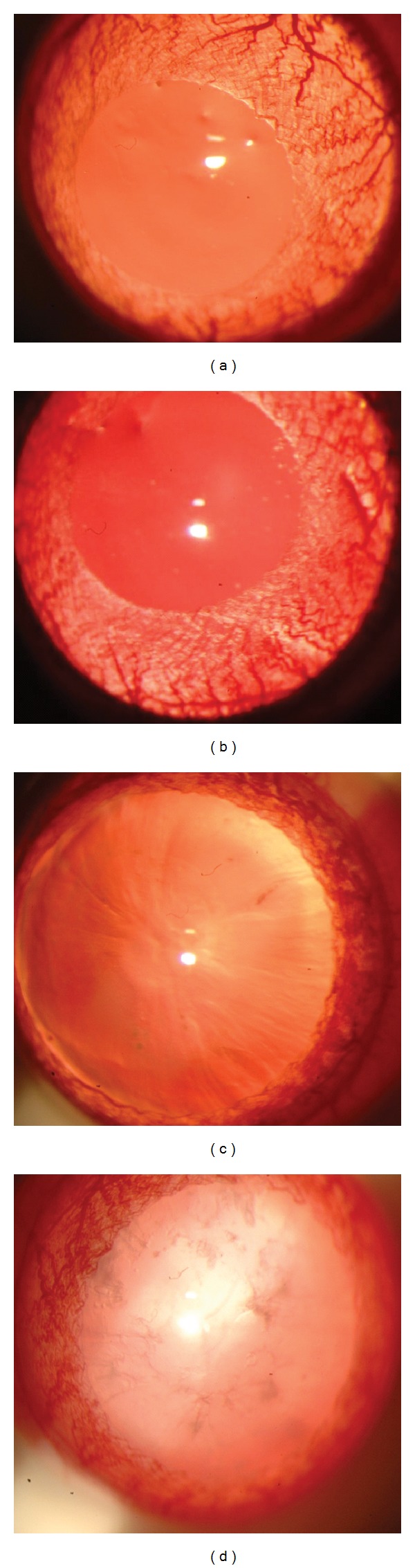
Representative photographs of cataract grading at the slit lamp for each group (a) control, (b) nicotine-treated, (c) diabetic, and (d) nicotine-treated diabetic. Both control and nicotine-treated groups (a and b) exhibit no observed changes and represent grade 0 according to the Oxford classification system. The diabetic group exhibits pronounced sutures and mild nuclear opacity. The nicotine-treated diabetic group displays prominent nuclear opacity.

**Figure 4:**
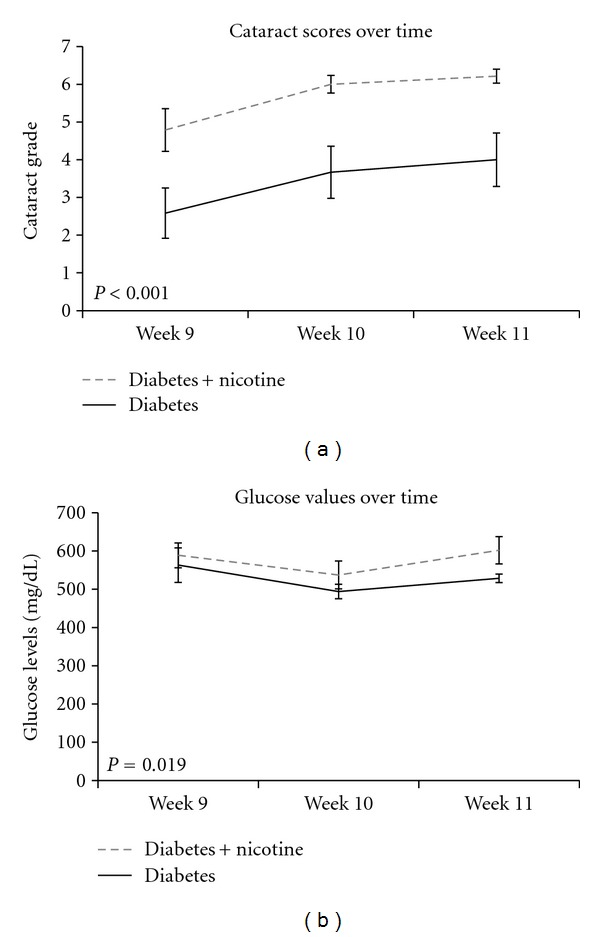
(a) Evolution of cataract scores over weeks 9, 10, and 11 of the study shows significantly higher scores for nicotine-treated diabetic rats (dashed line) compared to untreated diabetics (solid line) at each time point (*P* < 0.001). (b) Evolution of non-fasting blood glucose levels over weeks 9, 10, and 11 of the study shows significantly increased blood glucose levels for nicotine-treated diabetics (dashed) compared to their untreated counterparts (solid) (*P* = 0.019).

Lens opacities were assessed at 3 and 6 weeks but no significant change was seen between the nicotine-treated groups and the nontreated groups (data not shown). However, after 9 weeks of treatment, nicotine-treated diabetic rats had increased cataract scores as compared to untreated diabetics (*P* < 0.05) (Figure 2). The mean cataract severity for the untreated diabetic animals was 2.9, while it was 4.9 for the nicotine-treated diabetic rats. Representative images of lens opacities from each treatment group are shown in Figure 3. In a subset of animals, cataract grading was repeated at 10 and 11 weeks of nicotine treatment (experiment 2). Cataract scores continued to be greater in the nicotine-treated versus untreated diabetic groups at 10 and 11 weeks (*P* < 0.05). The control group treated with nicotine did not develop cataracts and did not differ from the untreated control group. In repeat experiment 2, we performed additional two-way ANOVA analysis to compare changes in glucose and cataract score over time (week 9 to 11) between groups. Cataract scores showed a significant increase over time in nicotine-treated versus untreated diabetics (*P* < 0.001) (Figure 4(a)), concomitant with a significant increase in glucose over time (*P* = 0.019) (Figure 4(b)).

### 3.3. Cytokines

**Figure 5:**
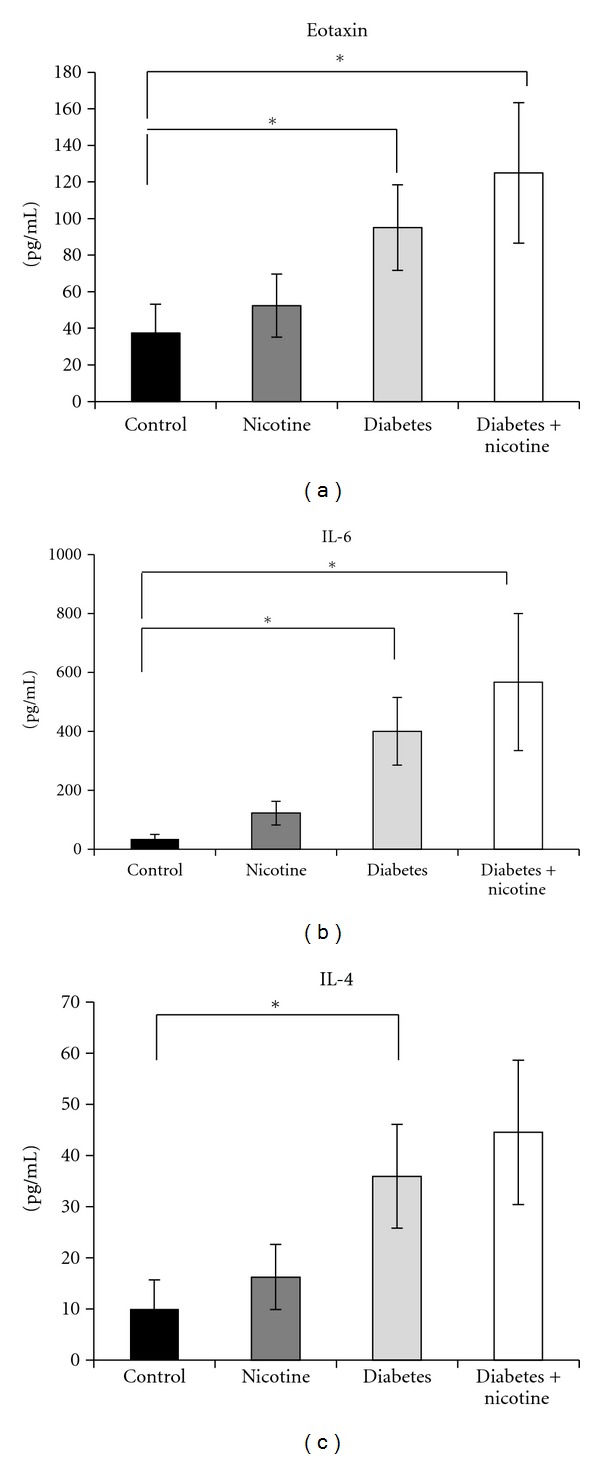
Rat serum cytokines are displayed at euthanasia. (a) There was a significant increase in serum Eotaxin between the diabetics and nicotine-treated diabetics as compared to control rats (*P* < 0.05). (b) There was a significant increase in serum IL-6 for the diabetic and nicotine-treated diabetic groups as compared to control rats (*P* < 0.05). (c) There was an increase in serum IL-4 in the diabetic group as compared to control (*P* < 0.05) and a nonsignificant positive trend in the nicotine-treated diabetic group. Significance base on one-way ANOVA with multiple comparisons versus control group (Dunn's method) (*P < 0.05, mean ± SEM).

Serum cytokine measurements showed a statistically significant increase in Eotaxin between nicotine-treated diabetics (125 ± 38 pg/mL) and untreated diabetics (95 ± 23 pg/mL) versus controls (37.3 ± 15.9 pg/mL) (Figure 5(a), *P* < 0.05). Additionally, we measured a significant increase in IL-6 levels in treated (567 ± 232 pg/mL) and untreated diabetics (400 ± 115 pg/mL) versus controls (34.4 ± 15.1 pg/mL) (Figure 5(b), *P* < 0.05). IL-4 levels increased significantly in the diabetic group (34.9 ± 10 pg/mL) (Figure 5(c), *P* < 0.05) and exhibited a non-significant positive trend in the nicotine-treated diabetic group (44.5 ± 14 pg/mL) versus controls (10 ± 5.6 pg/mL). 

## 4. Discussion

An epidemiological association between cigarette smoking and the development of cataracts has been well recognized [7–11, 20–22]. In addition, diabetes is a risk factor for cataract development [1–6]. However, the combined effect of nicotine and diabetes on cataract development has not been fully investigated. In this study, we examined the effects of nicotine on diabetes-induced cataract formation in an animal model. We observed an increase in severity of cataracts among nicotine-treated diabetic rats as compared to the untreated diabetic rats. Interestingly, there was no impact on cataract formation in the nicotine-treated control group within the time frame of our study (data not shown). The fact that nicotine increases the severity of cataracts in the treated diabetic group without affecting the treated controls suggests a potential synergistic mechanism(s) that exacerbates cataracts in diabetes. 

Although the mechanism by which nicotine promotes cataract development in diabetic rats is not known, nicotine causes oxidative stress and generates reactive oxygen species (ROSs) including superoxide and hydrogen peroxide [23]. In the case of diabetic rats, the lens fibers swell secondary to accumulation of polyols from excess sugars. Nicotine-induced ROS can interact with the lens protein and lipids, causing further damage to the already compromised lens fibers, leading to intense cataracts. Also, ROS and oxidative stress may inhibit the cellular antioxidants and inhibit the activity of antioxidant enzymes [24]. This can be inferred from the studies where it has been shown that dietary intake of antioxidants such as riboflavin, vitamins C, E, and carotene has an inhibitory effect on cataract formation [10]. Additionally, reduced glutathione replacement demonstrated protective properties in the context of nicotine-induced oxidative damage [25]. 

Systemic hyperglycemia and diabetes in rats have been associated with development of lens cortical lesions. The distribution of lesions has also been correlated to glucose transporter protein isoform 1 and 3 (GLUT 1, GLUT 3) distribution, linking cataract morphology to perturbations in glucose metabolism [26]. The systemic effect of nicotine on glucose uptake has been examined in dogs, rats [27], rabbits [28], and human volunteers [29]. The studies agree on a short-lived catecholinergic effect on the small intestines, resulting in an increase of glucose absorption, leading to an increase of blood insulin levels. This phenomenon is induced through both beta and alpha adrenergic receptors [30]. Pharmacological blockade of these receptors reduced nicotine-induced hyperglycemia [31]. Recently, Morgan et al. [29] described transdermal nicotine therapy leading to mild hyperglycemia in otherwise healthy patients, this in the setting of normal insulin and cortisol level. Therefore, nicotine may further disrupt glucose homeostasis in the diabetic environment thereby accelerating cataract development via a synergistic mechanism. 

The mechanism of cataract formation may also be dependent on inflammatory mediators. Increased IL-6 has been independently associated with both nicotine administration and cataract formation. In one such study, there was an increase in nuclear sclerotic cataracts associated with increased levels of serum IL-6 in elderly human subjects [32]. IL-6 elevation has also been characterized in serum and aqueous humor of diabetic patients [33]. Similarly, it has been reported that the concentration of IL-6 produced by peripheral blood mononuclear cells is increased briefly in smokers as compared to nonsmokers [34]. Our studies confirm elevated IL-6 levels in both diabetic and nicotine-treated diabetic animals. Furthermore, Mabley et al. [35] reported tissue-specific increases of IL-4 in animals treated with multiple low-dose streptozotocin (MLDS) and nicotine, as compared to their MLDS-treated counterparts. 

Eotaxin is a chemokine that has been implicated in numerous inflammatory disorders and may cause damage to healthy tissue by selectively recruiting eosinophils which release toxic proteins and oxygen radicals [36]. Lange et al. [37] demonstrated a significant increase in levels of eotaxin in vitreous samples from patients with proliferative diabetic retinopathy. Additionally, this study demonstrated a nearly 30% increase in plasma levels of eotaxin in diabetic patients compared to controls; however, these results failed to reach a statistical significance. Interestingly, no increase in eotaxin levels was detected in serum samples from patients with a genetic predisposition for developing type 1 diabetes [38]. We observed significant increases in eotaxin levels in both nicotine-treated and untreated diabetic groups which may be useful as a biomarker to evaluate ocular complications due to type 1 diabetes. 

In conclusion, the results of the current study suggest that nicotine exacerbates the development of cataracts in diabetic rats. Although the exact mechanism of action is unknown, a combination of oxidative stress and inflammation may play a key role.
